# Effect of sub-hypothermia blood purification technique in cardiac shock after valvular disease surgery

**DOI:** 10.1097/MD.0000000000019476

**Published:** 2020-03-27

**Authors:** Jihui Fang, Ming Xu, Bin Liu, Bo Wang, Haibo Ren, Haitao Yang, Yaling Dong, Laichun Song, Hongyan Xiao

**Affiliations:** a Department of Cardiac Surgery; b Department of Intensive Care Unit, Wuhan Asia Heart Hospital, Wuhan; c Department of Urinary Surgery, Dongfeng Maojian Hospital, Shiyan; d Department of Cardiology, Wuhan Asia Heart Hospital; e Department of Intensive Care Unit, Asia Heart Hospital, Wuhan University of Science and Technology, Hankou District, Wuhan, P.R. China.

**Keywords:** blood purification, cardiac surgery, cardiogenic shock, hypothermia

## Abstract

To observe the effect of sub-hypothermia (HT) blood purification technique in the treatment of cardiac shock after heart valve disease.

The patients were randomly divided into normothermic (NT) continuous blood purification (CBP) group (NT group) and HT CBP group (HT group). Observe the cardiac index (CI), the oxygen delivery (DO_2_) and oxygen consumption (VO_2_) ratio, Acute Physiology and Chronic Health Evaluation III(APACHE III) score, multiple organ dysfunction syndrome (MODS) score, dynamic monitoring of electrocardiograph, blood loss with or without muscle tremors, intensive care unit stay, mechanical ventilation time, CBP time, and the cases of infection and mortality at 0 day, 1 day, 2 day, 3 day; all above indicators were compared between 2 groups.

Ninety-five patients were randomly assigned into HT group (48 cases) and NT group (47 cases); there were no significant differences between the 2 groups for age, gender, pre-operative cardiac function, cardiothoracic ratio, and type of valve replacement (*P* > .05). There were no significant differences among the 1 day, 2 day, 3 day after recruited for CI, DO_2_/VO_2_ ratio, APACHE III score, MODS score (*P* > .05). But in HT group, DO_2_/VO_2_ ratio had been significantly improved after treatment for 1 day (2.5 ± 0.7 vs 1.8 ± 0.4, *P* = .024), and CI (3.0 ± 0.5 vs 1.9 ± 0.7, *P* = .004), APACHE III score (50.6 ± 6.2 vs 77.5 ± 5.5 *P* = .022), MODS score (6.0 ± 1.5 vs 9.3 ± 3.4, *P* = .013) also had been significantly improved after treatment for 3 days. In clinical outcomes, there were no significant differences between 2 groups for blood loss (617.0 ± 60.7 ml vs 550.9 ± 85.2 ml, *P* = .203), infection ratio (54.17% vs 53.19%, *P* = .341), the incidence of ventricular arrhythmia (31.25% vs 36.17%, *P* = .237), and muscle tremors (14.58% vs 8.51%, *P* = .346), while there were significant differences between 2 groups for intensive care unit stay (6.9 ± 3.4 days vs 12.5 ± 3.5 days, *P* = .017,), mechanical ventilation time (4.2 ± 1.3 days vs 7.5 ± 2.7 days, *P* = .034,), CBP time (4.6 ± 1.4 days vs 10.5 ± 4.0 days, *P* = .019), mortality (12.50% vs 23.40%, *P* = .024). But the incidence of bradycardia in HT group was much higher than the NT group (29.16% vs 14.89%, *P* = .029).

HT blood purification is a safer and more effective treatment than NT blood purification for patients who suffered from cardiac shock after valve surgery.

## 1 Introduction

Cardiac shock caused by cardiac pump failure is a common and serious complication during the peri-operative period of valve surgery, which can easily lead to multiple-organ dysfunction and extremely high mortality. Correcting the imbalance of oxygen supply and demand promptly is the key to reduce the mortality rate of cardiac shock after surgery. However, it is very difficult to increase oxygen delivery in patients after cardiac shock. Therefore, the clinical method of reducing oxygen consumption by means of sub-hypothermia (HT) can make the 2 tend to balance. The purpose of this study was to compare the clinical effects and complications of different temperature blood purification treatment on patients with cardiac shock.

## 2 Materials and methods

### 2.1 Patient selection and grouping

A prospective randomized case-control and non-blind method was used to select patients who had undergone heart valve disease replacement in our hospital from January 2011 to December 2014. Inclusion criteria were as follows:

1. Optional heart valve replacement surgery;
2. Patients had cardiac shock postoperative. The diagnostic criteria for cardiac shock were as follows: blood pressure < 90/60 mmHg and cardiac index <2.5 L/min.m^2^;
3. Patients were monitored by pulmonary artery floating catheter;
4. According to the “blood purification standard operating procedure” parallel continuous blood purification (CBP) treatment.

Exclusion criteria:

1. Pre-operative coronary atherosclerotic heart disease or peri-operative myocardial ischemia;
2. Patients had secondary surgical trauma;

Patients and their families were not willing to accept this clinical study. Cases that met the above criteria were divided into the normothermic (NT) group and HT group based on a random number table and an envelope concealment method.

Removal criteria:

1. Death within 72 hours after selection;
2. In addition to mechanical ventilation and blood purification, other mechanical assistances are also accepted.

The study was in line with medical ethics and approved by the Hospital Ethical Committee.

### 2.2 Treatment methods

All patients were monitored in intensive care unit (ICU), continuous analgesic sedative and ventilator-assisted ventilation, continuous monitoring of cardiogram, blood pressure, blood oxygen saturation, and central venous pressure using the Philip Monitor (Netherlands). And pulmonary arterial floating catheter grafting (Edwards Lifesciences^TM^ Vigilance II, CA) monitoring cardiac index, pulmonary artery wedge pressure, oxygen delivery, and oxygen consumption; counting the amount of urine and bleeding per hour; blood cell numbers were monitored and biochemical analyses were performed daily. Using antibiotics to prevent infection, proton pump inhibitors to prevent acute gastric mucosal lesions. Surgical issues resulting in unstable circulation were resolved. According to the patients’ condition, vascular active drugs were administered by micropump and hemoglobin was maintained at about 100 g/L. Blood purification was carried out at the bedside for patients who met the blood purification standard.^[1]^ Blood purification adopted continuous veno-venous blood filtration mode. The replacement fluid adopted an improved Port formula, pre-diluted, no anticoagulant. The volume of replacement fluid was 35 mL/kg.h with blood flow at 200 mL/min. Catheter was placed through the femoral vein using the Seldinger technique, using GAMBRO-PRISMAFLEX blood filter, CM-100 polysulfone membrane blood filter (Sweden) for 24 hours without interruption. In addition to the temperature, the treatment in the 2 groups was the same. The NT group used a blood filter to raise the temperature, maintained the temperature at 36.5°C to 37.3°C. In the HT group, the blood tube was not heated or placed in ice water and the temperature maintained at 34.0°C to 35.0°C.

### 2.3 Observation parameters

The 2 groups were statistically analyzed for CI, DO_2_/VO_2_ ratio, APACHE III score, and MODS score on the day of patient selection after 1, 2, and 3 days. The number of cases with bradycardia, ventricular arrhythmia, and muscle tremors were noted. In addition, ICU duration, mechanical ventilation time, CBP time, infection rate, blood loss, and mortality in the 2 groups were monitored.

### 2.4 Statistical methods

Enumeration data were expressed by rates, and *x*^*2*^ test was used to compare the data between the 2 groups. Measurement data were expressed as 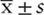, and *t* test was used to compare data between the 2 groups. All data were processed by SPSS l9.0 statistical software. *P* < .05 means significant difference.

## 3 Results

A total of 105 patients were enrolled, 4 of whom died within 72 hours after enrollment. Five of them received intra-aortic balloon pump, and 1 of them received extracorporeal membrane oxygenation. A total of 95 cases were eventually involved in the experiment, including 47 in the NT group and 48 in the HT group. There was no significant difference between the 2 groups for each index when enrolled (*P* > .05) (shown in Table 1). CI, DO_2_/VO_2_ ratio, APACHE III score, and MODS score at 1 day, 2 days, and 3 days were not significantly different in the NT group (*P* > . 05) compared with enrolled. In the HT group, the DO_2_/VO_2_ ratio was significantly improved (*P* < .05), but there were no significant differences in CI, APACHE III score, and MODS score at 1 day after treatment (*P* > . 05). There were significant differences in CI, APACHE III score, and MODS score between the 2 groups at 3 days after treatment compared to before treatment (*P* < . 05) (shown in Table 2). ICU residence time, mechanical ventilation time, CBP time, and the death rate in the HT group were all less than NT group and had statistical significance (*P* < .05) (shown in Table 3). There was no significant difference between the 2 groups about infection, ventricular arrhythmia, muscle tremors, and blood loss (*P* > .05), but the incidence of bradycardia in the HT group was higher than in the NT group (*P* < . 05) (shown in Table 4).

**Table 1:**
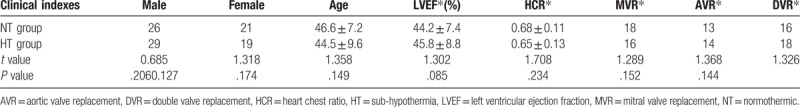
The general data of NT∗ group and HT∗ group.

**Table 2:**
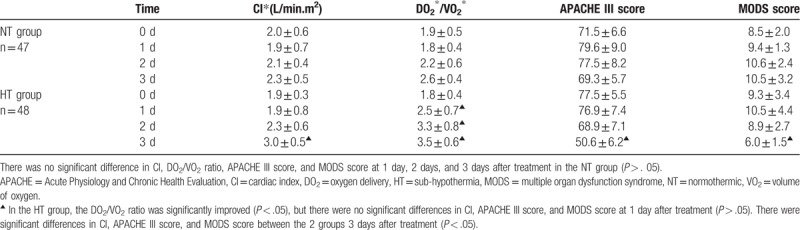
Clinical observation indices between NT∗ group and HT∗ group at different points in time (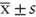).

**Table 3:**
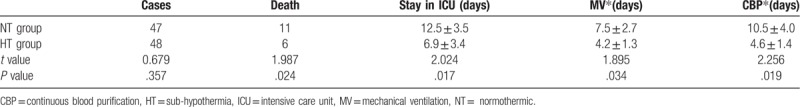
Comparison of clinical prognosis between NT∗ group and HT∗ group.

**Table 4:**
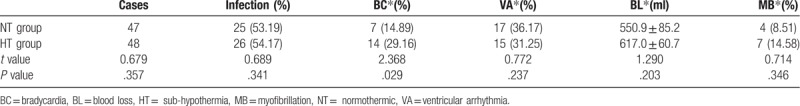
Comparison of complications between NT∗ group and HT∗ group.

## 4 Discussion

It has been reported that peri-operative death rate of severe valvular disease is as high as 11.9%,^[2]^ and postoperative cardiac shock is the main cause of death after cardiac valve replacement.^[3,4]^ Through mechanical assistance, heart failure can be recovered after cardiac surgery. That's probably because myocardial structure and metabolism has changed in a short-term of myocardial ischemia after cardiopulmonary bypass, but it has not yet necrosis. It causes its contraction function to return to normal for hours, days, or weeks after reperfusion.^[5]^ CBP has definite indications for cardiac shock after cardiac surgery. It can quickly correct the internal environmental disorders during shock, reduce the precardiac load through negative equilibrium, remove the molecular inflammatory medium and cardiac inhibitory factor, and improve heart function. In clinical practice, most CBP need to take measures to restore the blood temperature in the circulating pipeline to near normal human temperature before it is returned to the body. In this study, patients in the NT group were heated with a blood filter and maintain a temperature of 36.5°C to 37.3°C. The statistical analysis showed that there was no statistical difference for CI, DO_2_/VO_2_ ratio, APACHE III score, and MODS score at days 1 to 3 after treatment (*P* > .05). It indicated that CBP at normal temperature did not improve the imbalance of oxygen supply and demand. However, the temperature of CBP was maintained at 34.0°C to 35.0°C in the HT group, The DO_2_/VO_2_ ratio improved significantly on the first day after treatment (*P* < .05); the CI, APACHE III score, and MODS score improved 3 days after treatment compared to before treatment; and there were significant differences between the 2 groups (*P* < .05).

When the body temperature rises by 1°C, the body's basic metabolism increases by 13%.^[6]^ While the surgical trauma results in a dramatic increase in oxygen consumption. At this time, the new DO_2_/VO_2_ balance could be achieved under conditions of decreasing temperature without the increase workload to the heart. HT therapy has been recommended in guideline.^[7]^ Some studies about animal have suggested that HT could improve cardiac dysfunction after recovery,^[8,9]^ reduce myocardial injury after reperfusion in acute myocardial infarction, reduce the release of myocardial enzymes, reduce the myocardial infarction range, and increase the number of surviving myocardium in endangered areas.^[10]^ At the same time, studies have shown that HT has a protective effect on lung tissue during systemic inflammatory reactions and reduces the incidence of acute lung injury.^[11]^ In this study, the HT group combined CBP with HT to reduce the pre-cardiac load through negative balance of CBP stabilize the internal environment, and control the blood temperature. The oxygen consumption of the body is reduced without increasing the workload of the heart. At the same time, it protects the myocardium, reduces myocardial damage, delays the progress of the disease, and acquires time for the recovery of cardiac function and clinical transition. That is consistent with Yoo et al's^[12]^ research.

Chill is a common complication of HT. Other adverse effects of HT^[13,14]^ include arrhythmia, coagulation disorder, and electrolyte disorder and the risk of infection and sepsis may also increase. Most current studies have demonstrated that maintaining the temperature is not less than 32°C, and most of these side effects can be avoided.^[15]^ In the HT group, sufficient analgesic sedation and muscle looseness were adopted. In addition, the continuous veno-venous blood filtration model of predilution and no anticoagulant was adopted. In addition to the relatively high incidence of bradycardia, HT treatment did not lead to increased ventricular arrhythmia, muscle tremors, and infection after surgery compared to the normal temperature group. There was no significant difference in blood loss between the 2 groups.

## 5 Limitations

Our study has several limitations. First, the limited numbers of patients and events prohibited extensive subgroup analysis and further investigation into the late mortality. Second, there was no subsequent follow-up in this study, and more time was needed to understand the performance. Finally, this is a single-center prospective study associated with all the typical limitations of such a study design.

## 6 Conclusions

Most side effects can be avoided during HT blood purification treatment if the temperature is controlled not less than 32°C, except for bradycardia. In conclusion, HT blood purification is a safer and more effective treatment than NT blood purification for patients who suffered cardiac shock after valve surgery.

## Author contributions

**Conceptualization:** Laichun Song.

**Formal analysis:** Yaling Dong.

**Funding acquisition:** Hongyan Xiao.

**Methodology:** Hongyan Xiao.

**Resources:** Bin Liu, Haibo Ren.

**Software:** Ming Xu.

**Validation:** Haitao Yang.

**Visualization:** Bo Wang.

**Writing – original draft:** Jihui Fang.

## References

[1] Nesrallah GE Mustafa RA Macrae J Canadian society of nephrology guidelines for the management of patients with ESRD treated with intensive hemodialysis. Am J Kidney Dis 2013;62:187–98. 23566638 10.1053/j.ajkd.2013.02.351

[2] Sarraf N Thalib L Hughes A Cross-clamp time is an independent predictor of monality and morbidity in low- and high-risk cardiac patients. Int J Surg 2011;9:104–9. 20965288 10.1016/j.ijsu.2010.10.007

[3] Babaev A Frederick PD Pasta DJ Trends in management and outcomes of patients with acute myocardial infarction complicated by cardiogenic shock. JAMA 2005;294:448–54. 16046651 10.1001/jama.294.4.448

[4] Lichter-Konecki U Nadkarni V Moudgil A Feasibility of adjunct therapeutic hypothermia treatment for hyperammonemia and encephalopathy due to urea cycle disorders and organic acid emias. Mol Genet Metab 2013;109:354–9. 23791307 10.1016/j.ymgme.2013.05.014

[5] Laky D Parascan L Candea V Myocardial stunning. Morphological studies in acute experimental ischemia and intraoperatory myocardial biopsies. Rom J Morphol Embryol 2008;49:153–8. 18516320

[6] Dietrichs ES Kondrative T Tveita T Milrinone ameliorates cardiac mechanical dysfunction after hypothermia in an intact rat model. Cryobiology 2014;69:361–6. 25224046 10.1016/j.cryobiol.2014.09.002

[7] Field JM Hazinski MF Sayre MR 2010 American Heart Association guideline for CPR and ECC. Part 12: Postresuscitation support. Circulation 2010;122:s640–56. 20956217 10.1161/CIRCULATIONAHA.110.970889

[8] Roberts BW Kilgannon JH Chansky ME Therapeutic hypothermia and vasopressor dependency after cardiac arrest. Resuscitation 2013;84:331–6. 22885092 10.1016/j.resuscitation.2012.07.029

[9] Skulec R Kovarnik T Dostalova G Induction of mild hypothermia in cardiac arrest survivors presenting with cardiogenic shock syndrome. Acta Anaesthesiol Scand 2008;52:188–94. 18005380 10.1111/j.1399-6576.2007.01510.x

[10] Schwartz BG Kloner RA Thomas JL Therapeutic hypothermia for acute myocardial infarction and cardiac arrest. Am J Cardiol 2012;110:461–6. 22541421 10.1016/j.amjcard.2012.03.048

[11] Hamamoto H Sakamoto H Leshnower BG Very mild hypothermia during ischaemia and reperfusion improves post infarction ventricular remodeling. Ann Thorac Surg 2009;87:172–7. 19101292 10.1016/j.athoracsur.2008.08.015 PMC3021243

[12] Yoo JS Kim JB Jung SH Surgical repair of descending thoracic and thoracoabdominal aortic aneurysm involving the distal arch: open proximal anastomosis under deep hypothermia versus arch clamping technique. J Thorac Cardiovasc Surg 2014;148:2101–7. 25131174 10.1016/j.jtcvs.2014.06.068

[13] Raza S Ali Baig M Chang C A prospective study on red blood cell transfusion related hyperkalemia in critically ill patients. J Clin Med Res 2015;7:417–21. 25883703 10.14740/jocmr2123w PMC4394913

[14] Bernard SA Buist M Induced hypothermia in critical care medicine: a review. Crit Care Med 2003;31:2041–51. 12847402 10.1097/01.CCM.0000069731.18472.61

[15] Lopez-de-Sa E Rey JR Armada E Hypothermia in comatose survivors from out-of-hospital cardiac arrest: pilot trial comparing 2 levels of target temperature. Circulation 2012;126:2826–33. 23136160 10.1161/CIRCULATIONAHA.112.136408

